# Variable coloration is associated with dampened population fluctuations in noctuid moths

**DOI:** 10.1098/rspb.2014.2922

**Published:** 2015-06-07

**Authors:** Anders Forsman, Per-Eric Betzholtz, Markus Franzén

**Affiliations:** 1 Center for Ecology and Evolution in Microbial Model Systems, EEMiS, Department of Biology and Environmental Science, Linnaeus University, 39182 Kalmar, Sweden; 2 Department of Biology and Environmental Science, Linnaeus University, 39182 Kalmar, Sweden; 3 Department of Community Ecology, UFZ, Helmholtz Centre for Environmental Research, Halle, Germany

**Keywords:** colour polymorphism, dynamics, ecological diversity, moths, noctuidae, population fluctuations

## Abstract

Theory and recent reviews state that greater genetic and phenotypic variation should be beneficial for population abundance and stability. Experimental evaluations of this prediction are rare, of short duration and conducted under controlled environmental settings. The question whether greater diversity in functionally important traits stabilizes populations under more complex ecological conditions in the wild has not been systematically evaluated. Moths are mainly nocturnal, with a large variation in colour patterns among species, and constitute an important food source for many types of organisms. Here, we report the results of a long-term (2003–2013) monitoring study of 115 100 noctuid moths from 246 species. Analysis of time-series data provide rare evidence that species with higher levels of inter-individual variation in colour pattern have higher average abundances and undergo smaller between-year fluctuations compared with species having less variable colour patterns. The signature of interspecific temporal synchronization of abundance fluctuations was weak, suggesting that the dynamics were driven by species-specific biotic interactions rather than by some common, density-independent factor(s). We conclude that individual variation in colour patterns dampens population abundance fluctuations, and suggest that this may partly reflect that colour pattern polymorphism provides protection from visually oriented predators and parasitoids.

## 1. Introduction

Exploring the causes and consequences of population dynamics of species in the wild is key to better understanding of evolution and maintenance of biodiversity. Smaller populations may be more extinction prone due to demographic stochasticity and Allee effects [1–4], and population fluctuations can increase extinction risk via the eroding effect of small population size and bottlenecks on genetic diversity [3,5–9]. Larger and more productive populations can function as ‘source’ areas, provide immigrants that can have a rescuing effect, prevent extinction of populations in adjacent ‘sink’ areas with negative growth, (re-)colonize locally extinct populations and establish new populations in previously unoccupied areas [10,11]. Identifying shared ecological characteristics that influence abundance fluctuations is thus crucial for a better understanding of local extinctions, recolonizations and range distribution shifts, and can aid the development of conservation management and pest control.

Here, we address the hypothesis that greater inter-individual genetic and phenotypic variability should shield against environmental change, enable faster growth, allow for higher densities and reduce fluctuations in population size [12–17]. Experimental investigations into the role of diversity for population fluctuations are rare, typically of short duration and conducted under controlled, relatively simple environmental conditions [18–22]. The results of manipulation experiments can vary depending on ecological settings [23]; for instance, diversity has been found to exert stronger effects on population performance in more stressful or complex natural environments than under simplified laboratory conditions [24]. Given the impact of abundance fluctuations on population viability, there is a need for more studies that address this issue under natural conditions in the wild.

**Figure 1.**
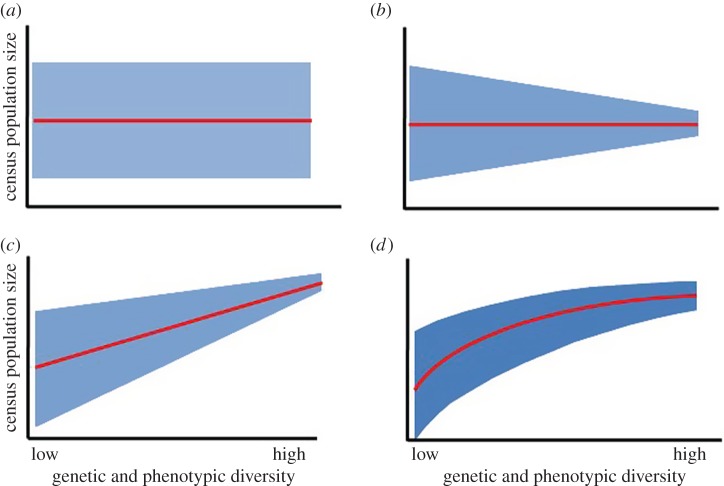
Illustrations of how genetic and phenotypic diversity (inter-individual variation) may affect population dynamics by influencing (arithmetic) mean and variance in census population size. The four outcomes shown are: (*a*) increasing diversity affects neither mean performance nor the variance (null hypothesis of no effect); (*b*) increasing diversity does not affect mean performance but reduces the variance; (*c*) increasing diversity results in a linear increase in mean performance and reduces the variance, and (*d*) increasing diversity results in a curvilinear asymptotic increase in mean performance and reduces the variance. (Online version in colour.)

In addition to establishing whether inter-individual diversity is important, it is imperative to determine the shape of the relationship linking population performance to inter-individual diversity [8]. At least four major outcomes are possible: (i) increasing diversity affects neither mean performance nor the variance (that is the null hypothesis of no effect); (ii) increasing diversity does not affect the mean performance but reduces the variance (e.g. [20]); and (iii) increasing diversity results in a linear or (iv) curvilinear (asymptotic) increase in mean performance, and reduces the variance (e.g. [25]; figure 1*a–d*). In the context of biodiversity conservation, where there are many other practical constraints, different outcomes call for different conservation measures. For instance, if the relationship follows the law of diminishing returns, it may be more rewarding beyond a certain point to redirect resources to meet other needs than under a linear ‘more is better’ scenario (see also Discussion).

We use long-term (2003–2013) abundance data for 246 species of noctuid moths having non-variable, variable or highly variable colour patterns to examine whether inter-individual diversity dampens population fluctuations, and to investigate the shape of the relationship linking diversity to population performance. There are several mechanisms by which increased individual variation in colour pattern can influence fitness and stabilize population dynamics [15]. For example, colour patterns can provide camouflage or function as warning signals [26], and colour polymorphism may protect prey populations from predators by reducing their search performance and lowering perceived prey density [27–30]. Colour patterns may also play a role in intraspecific signalling [31,32]. In many animals, colour patterns are associated with physiological, morphological, behavioural and life-history phenotypic dimensions [33,34]. Darker pigmentation may protect against harmful ultraviolet radiation [35], and can impact healing capacity, cellular innate immunity and parasite resistance [36,37]. Furthermore, individuals with pale and dark colour patterns have different heat balance [38,39]. In ectothermic animals, such as insects and reptiles, effects of coloration mediated via body temperature on individual microhabitat use, physiology, activity patterns and performance can therefore be important [38–43]. When colour pattern is associated with fitness, abundance fluctuations can also be mediated by natural selection causing changes in the frequency distribution of phenotypes that differ in reproductive capacity [44–47].

Colour polymorphism may be accompanied by differential utilization of food resources [48], and such niche partitioning can reduce intraspecific competition [49,50]. A more efficient exploitation of a greater diversity of resources (a broader niche when summed over different morphs or groups of individual specialists) represents one of the key mechanisms envisaged to positively influence growth, stability and persistence of polymorphic populations [14–16,50,51]. Finally, genetically and phenotypically more diverse groups and populations are more likely (sampling effect) to harbour more productive or pre-adapted phenotypes [20,25], and this may have a variance-reducing, bet-hedging effect that increases population fitness [52].

## 2. Material and methods

### (a) Study area, moth survey and quantification of abundance fluctuations

Moths lend themselves admirably to comparative investigations of how colour pattern variation influences population performance. Chromatic polymorphism has evolved and been lost independently in a series of distantly related lineages of moths (cf. [53]). Because they are largely nocturnal and attracted to artificial light sources, their abundance fluctuations can be monitored over long time periods using standardized procedures [53–55]. Furthermore, moths constitute a large proportion (approx. 135 000 thousand out of 155 000 species [56]) of the Lepidoptera [53,57], are an important food source for many birds, bats, lizards and spiders, use a broad range of host-plant species, and act as pollinators. Some species of moths are endangered and the focus of conservation concerns [57,58], whereas others are economically important pest species in agricultural and forest ecosystems [57,59]. It is therefore dissatisfying that relatively little is known about the patterns and causes of abundance fluctuations, local extinctions and recolonizations in moths [54,55,57,60].

The monitoring study was established near the village of Böste (55°20′37″ N, 13°18′54″ E) in the southernmost part of Sweden. The area is dominated by agriculture (more than 75% arable land) that is typical for this region. The remaining quarter consists of scattered grasslands, meadows, mixed forests and built-up areas. The trap was placed outside Böste (100 inhabitants) on the edge between a garden, arable field and grassland, and it remained at the same place throughout the study. Placing the trap at a three-way ecotone is a good way to maximize the number of species. To monitor moth abundance, we used an automatic Ryrholm light-trap [61] with a mercury vapour lamp (125 W Hg-bulb). Noctuid moths have a rather homogeneous attraction to mercury lamp sources [53]. Light-traps can be operated during all the hours of darkness, almost regardless of weather, and thus are suitable for monitoring abundance patterns [62]. In our study, the light-trap was in operation during 11 years (2003–2013), from March to November. The trap was checked and emptied every third week. Jonason *et al*. [62] analyse data from one trap collecting every night over 1 year and show that a single trap gives robust data of both abundance and species richness. In our study, each specimen was counted and determined to species level. The total number of individuals captured of each species in each year was used as a measure of annual abundance (census population size). The coefficient of variation (CV) of annual abundance across the 11 years was used as a measure of fluctuations [63].

### (b) Study species, classification of colour pattern diversity, host-plant specificity and activity period

Two authors (P.E.B. and M.F.) performed expert classifications, independently of each other and blind with regard to abundance data, of colour pattern variability of all noctuid species recorded in the trap survey (*n* = 246 species). Species within which there is no apparent variation among individuals in coloration and wing pattern were classified as non-variable; species in which individuals vary in the size, shape or coloration of colour pattern elements were classified as variable; and species in which individuals vary considerably in size, shape and coloration of pattern elements or with regard to presence/absence of pattern elements were classified as highly variable. We classified species that were sexually dichromatic as having variable coloration only if variation was manifest within one or both sexes, otherwise they were considered non-variable. The independent classifications diverged for only six (2.4%) of the 246 species, and in these cases we consulted Skou [64] before the final classification was made. The classification of species with regard to colour pattern variability used in the analyses was thus very robust.

Moths differ in the degree of host-plant specificity, and species with narrower feeding niches may have lower abundances [51,55,65]. If species that have monophagous larvae also have less variable colour patterns as adults, an association of population abundance with variable coloration might be driven by a confounding underlying effect of host-plant specificity, rather than by mechanisms that involve coloration. To evaluate this possibility, all species were classified for niche breadth as belonging to one of three categories of larval host-plant specificity: monophagous species, which feed only on a single plant species; oligophagous species, which feed on a few plants species (use between two and five species or restricted to a particular plant genera/family); and polyphagous species, which feed on six or more different plant species or genera [55].

If colour pattern variation of adults is influenced by seasonality and temperature, adults of spring- and autumn-emerging species might have more variable colour patterns and also undergo greater (or smaller) abundance fluctuations due to differences in the constancy of weather conditions. To evaluate this possibility, all species were classified as belonging to one of four classes of flight activity period: spring (March–May), summer (June–August), autumn (September–November) or autumn and spring (September–April), according to Svensson [66].

A list of species included in our dataset and their classification with regard to colour pattern variation, host-plant specificity and activity period is available in the electronic supplementary material, table S1.

### (c) Statistical analyses of data

To discriminate among potential drivers of the abundance fluctuations uncovered by our data (see below), we assessed the degree of interspecific synchrony. This was done to get an indication as to whether the dynamics are likely to be influenced more strongly by density-independent abiotic factors than by density-dependent biotic interactions [67–70]. It was expected that if some common, density-independent factor(s) such as annual variation in weather conditions was responsible for the dynamics, this would manifest as a high degree of synchrony in abundance fluctuations among species. By contrast, if species-specific biotic interactions with host plants, competitors, predators and parasites were the dominating drivers of abundance fluctuations, this would be indicated by low interspecific synchrony. To evaluate whether abundance fluctuations within species among the 11 years were correlated across species, and to test whether species were more synchronous than expected under a random null hypothesis, time-series data were analysed using Kendall's coefficient of concordance, *W*, based on rank-transformed annual abundance data [71,72]. Additionally, the average Spearman correlation coefficient (*r*_s_) on the ranks of all pairs of species was computed as the linear transformation of Kendall's *W*, as *r*_s_ = (*mW* − 1)/(*m* − 1), where *m* is number of species and *W* is Kendall's *W* [72].

Mean abundance was calculated for each species across the 11 sampling years, and abundance fluctuations were quantified and compared using CV of abundance over years [18,63]. Comparative analyses should ideally be performed based on highly resolved phylogenies that contain information on branch lengths, such that the directionality and order of state transitions can be estimated [73]. Unfortunately, such phylogenies are available only for a subset of Swedish moths. Furthermore, our abundance dataset comprises only a subset of all 430 noctuid species in Sweden (more than 42 000 species are known worldwide of Noctuid moths in more than 4800 genera [74]), and the results of phylogeny-based comparative analysis are sensitive to missing data in the phylogeny [75].

To test whether abundance and stability were associated with colour pattern diversity while statistically adjusting for variation among genera, we performed general linear mixed models (GLMMs) implemented using the procedure MIXED in SAS [76,77]. We analysed abundance and stability first separately and then together. In the univariate approach, we treated log-transformed species mean abundance computed over the 11-year sampling period as the dependent variable, colour pattern (non-variable, variable or highly variable) as a fixed factor with three levels, adult activity period as a fixed effect with four levels, larval niche breadth as a fixed effect with three levels, and genus was included as a random factor to account for greater similarity among more closely related species. To test for associations with abundance fluctuations in the univariate approach, we instead treated CV of abundance across the 11 years as the dependent variable. For both analyses, the Kenward–Roger method was used to approximate degrees of freedom [77]. Statistical significance of fixed effects was assessed using *F*-statistic in the Type 3 test for fixed effects [76]. Statistical significance of random factors was assessed both using the Wald *Z*-test, and using the log-likelihood ratio test with one degree of freedom per random effect [76,77]. To evaluate whether the relationships linking colour pattern diversity to abundance and stability were linear or curvilinear, we performed quadratic regression analysis, and modelled colour pattern variability as a covariate (regressor), rather than as a fixed effect, using the MIXED procedure in SAS.

Because average population density and the magnitude of density fluctuations can be correlated [78], we also tested for effects of colour pattern variability on abundance and stability using a multivariate repeated measures analysis of variance approach implemented with the MIXED procedure in SAS [76,79]. In this analysis, log mean abundance and CV of abundance across years for each species were treated as repeated response variables, colour pattern as a fixed factor, and species and genus were included as random factors. We used the maximum-likelihood method, an unstructured covariance matrix, and the Kenward–Roger approximation to determine the degrees of freedom [76].

### (d) Systematics of moths

The systematics and phylogenetic relationships of noctuid moths is subject to debate and under revision. For the results reported below, we used the systematics of Karsholt & Razowski [80]. According to a more recent systematic treatment [81], some of the species in our dataset have been renamed and assigned to different genera, and 15 species in our dataset have been moved from family Noctuidae to family Erebidae (see electronic supplementary material, table S1). To evaluate whether our results and conclusions are influenced to any important degree by the systematic arrangements of species, we analysed our data also using the more recent systematics of Karsholt & Stadel Nielsen [81], and we did this in two ways, both including and omitting the 15 Erebidae species. The rearrangement of some species and the reduction in sample size when omitting Erebidae species, *sensu* Karsholt & Stadel Nielsen [81], had the result that the exact parameter values of the statistical tests changed somewhat, but these minor changes did not in any way influence the results or conclusions regarding abundance and stability in relation to colour pattern variability (see the electronic supplementary material, supporting results).

## 3. Results

### (a) Abundance fluctuations and degree of interspecific temporal synchrony

**Figure 2.**
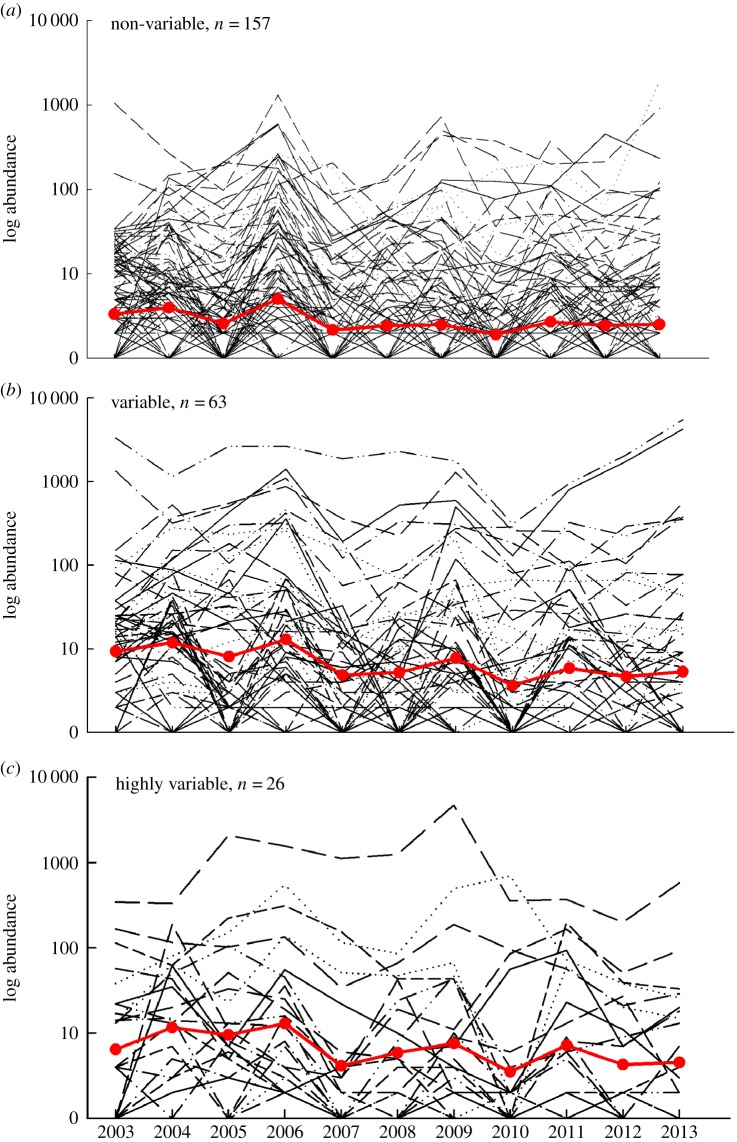
Time series showing raw data on population abundance fluctuations in noctuid moths with non-variable, variable or highly variable colour patterns. Each thin line shows data on annual abundance for one species over the period 2003–2013. Dots and thicker lines represent annual means calculated across species. For the statistical analyses, mean abundance was instead calculated within each species across the 11 years, and abundance fluctuation was estimated for each species as CV of abundance over years. Note that abundance data are shown on a logarithmic scale. (Online version in colour.)

During the 11-year field survey, 115 100 individual noctuid moths were captured, providing abundance data for 246 species distributed among 124 genera. Total capture rates ranged from 6425 to 17 325 (mean = 10 464) individuals per year. Abundances varied among species within years and among years within species (figure 2). The highest annual abundance (*n* = 5476 individuals) for a single species was recorded for *Xestia c-nigrum* in 2013.

Interspecific temporal synchrony in abundance fluctuations during the 11-year time series across all 246 species was low (Kendall's *W* = 0.07, *χ*^2^ = 172.79, d.f. = 10, *p* < 0.0001), and the average Spearman correlation coefficient on the ranks of all pairs of species was weak (*r*_s_ = 0.06).

### (b) Abundance and stability in relation to colour pattern variability

**Figure 3.**
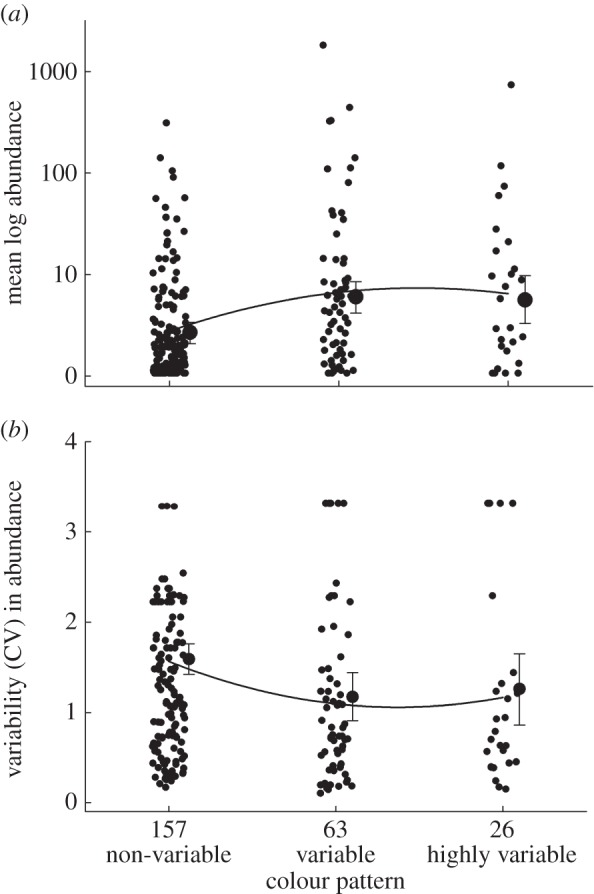
Comparisons of (*a*) average abundance and (*b*) among-year variability in abundance (measured as CV of the 11 annual abundance estimates) in noctuid moths with non-variable, variable and highly variable colour patterns. Individual species data points are shown (smaller dots to the left). Data points on abundance and variability in abundance have been jittered in the *x*-axis direction. Larger dots and error bars to the right represent means (±95% CL) as estimated from general linear mixed model analyses based on log-transformed species data with genus treated as random factor, and colour variability as a fixed factor. Results are based on 11 years (2003–2013) of abundance data for 246 moth species, representing 124 genera. Note that abundance data are shown on a logarithmic scale. Numbers below horizontal axis denote sample sizes. To normalize distributions and homogenize variances, data in the graphs were log-transformed prior to analyses.

Mean abundance varied significantly among noctuid genera and among the three levels of colour pattern diversity (general linear mixed model, GLMM, analysis, implemented using the procedure MIXED in SAS, applied to log-transformed species means computed over the 11-year sampling period, random effect of genus: estimate = 0.054 ± 0.0276 s.e., log-likelihood ratio *χ*^2^ = 4.4, d.f. = 1, 0.025 < *p* < 0.5, Wald *Z* = 1.95, *p* = 0.025; Type 3 test for fixed effect of colour pattern: *F*_2,236_ = 9.33, *p* = 0.0001; fixed effect of larval niche breadth: *F*_2,185_ = 0.33, *p* = 0.54; fixed effect of adult activity period: *F*_3,147_ = 1.36, *p* = 0.26). On average, species with highly variable (mean of annual abundances across species = 6.5 ± 1.5 s.d. individuals) or variable (6.6 ± 1.5 s.d.) colour patterns were about twice as abundant, compared with species having non-variable (2.7 ± 1.3 s.d.) colour patterns (figure 2*a–c*). Greater intraspecific variability in colour pattern was associated with higher abundance also after statistically adjusting for the variation in abundance among genera, and quadratic regression analysis demonstrated that the relationship linking mean abundance to colour pattern diversity was curvilinear (figure 3*a*).

Comparisons of among-year variability in abundance (estimated as within-species CV of the annual abundances) uncovered that species with non-variable colour patterns had greater fluctuations in abundance than did species with variable and highly variable colour patterns (GLMM, random effect of genus: estimate = 0.0315 ± 0.0715 s.e., log-likelihood ratio *χ*^2^ = 4.4, d.f. = 1, 0.025 < *p* < 0.05, Wald *Z* = 0.44, *p* = 0.33; fixed effect of colour: *F*_2,238_ = 5.15, *p* = 0.0064; fixed effect of larval niche breadth: *F*_2,238_ = 2.78, *p* = 0.064; fixed effect of activity period: *F*_3,238_ = 0.80, *p* = 0.49), and results from quadratic regression analysis uncovered that the relationship linking the magnitude of abundance fluctuations to diversity was nonlinear (figure 3*b*).

There was no indication that species having more variable colour patterns as adults used a greater (or lower) diversity of host-plant species (Mantel–Haenszel *χ*^2^ = 0.58, d.f. = 1, *p* = 0.45, *n* = 246 species). The degree of variation in wing colour pattern was independent of the timing of the species' adult flight period (Cochran–Mantel–Haenszel stratified test for a general association of colour variation and activity period after controlling for genus, *χ*^2^ = 6.18, d.f. = 6, *p* = 0.40). The pattern of higher average abundance and reduced between-year fluctuations in species with more variable colour patterns remained statistically significant when the potentially confounding effects of degree of larval host-plant specificity and of flight period were not adjusted for in the model (mean abundance: fixed effect of colour pattern: *F*_2,240_ = 8.90, *p* = 0.0002; CV of abundance: fixed effect of colour pattern: *F*_2,228_ = 4.26, *p* = 0.0153).

Our findings are robust to choice of statistical test; we arrive at the conclusion that colour pattern variability was weakly but statistically significantly associated with average abundance and fluctuations also when data were analysed using a multivariate analysis of variance approach (overall fixed effect of colour pattern, Wilks *Λ* = 0.92, *χ*^2^ = 21.7, d.f. = 4, *p* < 0.001).

## 4. Discussion

Do species with higher levels of among-individual diversity in functionally important traits—such as colour pattern—have higher average abundances and dampened population fluctuations compared with less diverse species, as predicted by theory [12–16]? Based on the analysis of 11-year time-series data for 246 species of noctuid moths, we found that species with greater levels of inter-individual variation in colour pattern have higher average abundances and undergo smaller between-year abundance fluctuations compared with species having less variable or non-variable colour patterns. Importantly, the association of abundance with colour pattern was evident also after we statistically controlled for the potential effects that variation in niche width (as quantified by number of host-plant species used) and adult flight period might have both on evolutionary modifications of colour pattern diversity and on the dynamics of population fluctuations. Our study further provides rare information on the nature of the relationship linking population fitness to the level of inter-individual variation, and specifically suggests that increasing diversity results in a curvilinear asymptotic increase in mean performance and a reduction of the variance in performance (cf. figure 1*d*).

### (a) Underlying mechanisms

Pinpointing the driving ecological interactions and mechanisms by which higher levels of colour pattern diversity may have contributed to higher and more stable population abundance goes beyond the scope of this study. However, the low degree of interspecific synchrony in abundance fluctuations points to the conclusion that species-specific biotic interactions, rather than some density-independent abiotic factor(s), were the dominating drivers of the abundance fluctuations in moths demonstrated here [67–70]. Of these, the role of colour pattern and polymorphism in providing protection from visually oriented predators and parasitoids [27–29,82,83] seem particularly plausible because moths are robustly built and able to fly only under high ambient temperatures, such that avoiding detection or averting attacks is crucial [30]. However, linkage density of interactions and connectance among species within natural food webs may be immensely more complex than anticipated based on direct observations of pairwise encounters [84], and this makes it difficult in most systems to identify the species and type of interaction of greatest importance for abundance fluctuations.

The association of variable colour pattern with abundance fluctuations may also reflect a role of behavioural, physiological or life-history traits linked to colour pattern variation [33,34]. Species traits of moths and butterflies have been studied in relation to range shifts [55,85–87], extinction risks [51,88], and responses to habitat area and isolation [65]. That a statistically significant signature of among-individual variation in colour pattern was apparent across this systematically heterogeneous group also after we statistically controlled for any potential effects of variation among species in niche breadth and flight period, and despite the fact that we did not statistically control for abundance variation associated with other potentially important species traits, suggests that colour pattern plays an important role.

We find it unlikely that unknown ‘cryptic’ species are hidden in our data; recent molecular studies show that most of the Noctuidae species in Scandinavia have been described and have the correct species status [89].

### (b) Implications for biodiversity conservation and invasive species management

As populations decline, combinations of biotic and abiotic processes can interactively drive populations to extinction [5,90]. Schoener *et al.* [9] studied the dynamics of extinction of island populations of spiders that had been monitored continuously over a 20-year period and report that long-term population growth rate is very sensitive to patterns of juvenile survivorship and to variation in the population ceiling and environmental noise, showing that sources of population fluctuations are important. Fagan & Holmes [3] analysed data for 10 vertebrate species (two mammals, five birds, two reptiles and a fish) whose final extinction was witnessed via monitoring, and report that the time to extinction scales to the logarithm of population size and that year-to-year variability in abundance was highest as populations approached extinction [3]. We are not aware of any previous comparative or experimental investigation in any group of animals into whether variable coloration influences abundance fluctuations. However, our present findings suggest that colour pattern variation may be used to help identify populations and species that are relatively more vulnerable to population fluctuations, and therefore ultimately may also face a greater risk of extinction (see also [91–96]).

Larger and more productive populations probably produce more dispersers during peak years, and thereby influence range expansions and outbreak dynamics [59]. Our present findings therefore add support to the notion [15,24,97] that incorporating colour pattern variability in the list of species characteristics used for identifying potentially harmful species could improve projections and future risk assessments.

Determining the shape of the relationship linking population fitness to inter-individual diversity (figure 1) is essential in management of biodiversity, where there are many other practical constraints, and where different outcomes call for different designs of conservation programmes [8]. Under a curvilinear asymptotic relationship with diminishing returns, as indicated by our present study and by the experimental results of Ellers *et al.* [98], efforts towards increasing diversity may initially be very rewarding while generating little additional benefit beyond a certain level of investment (cf. figure 1*d*). Under such conditions, redirecting resources to meet other needs may be more strategic than under linear ‘more is better’ scenarios. If increasing diversity contributes to reduced variance in performance and population fitness, as indicated by our present results and previous studies [20,25], this suggests that the incorporation of bet-hedging theory [52,99] may allow for better-informed decisions regarding how to best divide conservation resources and design management programmes.

## 5. Conclusion

In conclusion, our time-series data of noctuid moths provide rare evidence, in accordance with expectations from theory [12–17], that greater diversity among individuals in colour pattern is associated with increased average abundance and stability in the wild. Insights of the role of variation among individuals in functionally important traits for population dynamics may vitalize research, improve management plans for protection of endangered species, and enhance identification and control of economically important pest species in agricultural and forest ecosystems. Obtaining irrefutable evidence that greater levels of inter-individual diversity dampen population fluctuations is hampered by reciprocal influences between rapid evolution and ecological dynamics [100,101]. To combine experimental manipulation of the degree of colour pattern variation [96,102] with long-term monitoring of experimental populations may offer a tractable approach towards addressing this issue in the future.

## Supplementary Material

ESM Table S1 - 2015 04 14 reduced.pdf

## Supplementary Material

ESM Supporting Results 2015 04 14.pdf

## Data accessibility

Data are available in the electronic supplementary material.
