# Supplementary material for: Variable coloration is associated with dampened population fluctuations in noctuid moths
Source: Proc Biol Sci. 2015 Jun 7;282(1808):20142922. doi: 10.1098/rspb.2014.2922 (PMC4455791; doi:10.1098/rspb.2014.2922)
Supplement: ESM Table S1 - 2015 04 14 reduced.pdf [file rspb20142922supp1.pdf]

Electronic Supplementary Material ESM (for publication as supporting information on the Proc R Soc London B website)

Variable coloration is associated with dampened population fluctuations in noctuid moths

Anders Forsman, Per-Eric Betzholtz, Markus Franzén

[1] Author for correspondence E-mail: anders.forsman@lnu.se

Table S1. List of the 246 moth species for which annual abundance pattern data was collected from 2003 to 2013. Table shows taxomomy and systematics of the 246 moth species in our data set sensu Karsholt & Razowski 1996 [1] and sensu Karsholt & Stadel Nielsen 2013 [2]. Asterisks (*) denote that the species was moved from family Noctuidae to family Erebidae by Karsholt & Stadel Nielsen 2013. Each species was classified with regard to degree of inter-individual variation in colour pattern: non-variable (0), variable (1), or highly variable (2). Each species was classified for niche breadth as belonging to one of three categories of larval host-plant specificity: monophagous species that feed only on a single plant species, oligophagous species that feed on a few plants species (use between two and five species or restricted to a particular plant genera/family), and polyphagous species that feed on six or more different plant species or genera [3]. Each species was classified as belonging to one of four classes of flight activity period: spring (Sp, March-May), summer (Su, June-August), autumn (Au, September - November) or autumn and spring (AuSp, September-April), according to Svensson [4].

Genus and species according to Karlsholt & Razowski 1996 [1]	Genus and species according to Karsholt & Stadel Nielsen 2013 [2]	Colour pattern diversity	Host plant specificity	Flight period
Abrostola tripartita	Abrostola tripartita	0	mono	Su
Abrostola triplasia	Abrostola triplasia	0	mono	Su
Acronicta aceris	Acronicta aceris	0	poly	Su
Acronicta cuspis	Acronicta cuspis	0	poly	Su
Acronicta megacephala	Acronicta megacephala	0	poly	Su
Acronicta psi	Acronicta psi	0	poly	Su
Acronicta auricoma	Acronicta auricoma	1	poly	Su
Acronicta leporina	Acronicta leporina	1	poly	Su
Acronicta rumicis	Acronicta rumicis	1	poly	Su
Actinotia polyodon	Actinotia polyodon	0	mono	Su
Aetheria bicolorata	Hecatera bicolorata	2	oligo	Su
Agrochola circellaris	Agrochola circellaris	0	poly	Au
Agrochola helvola	Agrochola helvola	0	poly	Au
Agrochola litura	Agrochola litura	0	poly	Au
Agrochola lota	Agrochola lota	0	poly	Au
Agrochola nitida	Agrochola nitida	0	poly	Au
Agrochola lychnidis	Agrochola lychnidis	1	poly	Au
Agrochola macilenta	Agrochola macilenta	1	poly	Au
Agrotis clavis	Agrotis clavis	1	poly	Su
Agrotis exclamationis	Agrotis exclamationis	1	poly	Su

Agrotis ipsilon	Agrotis ipsilon	1	poly	Su
Agrotis ripae	Agrotis ripae	1	oligo	Su
Agrotis vestigialis	Agrotis vestigialis	1	poly	Su
Agrotis segetum	Agrotis segetum	2	poly	Au
Ammoconia caecimacula	Ammoconia caecimacula	0	poly	Au
Amphipoea fucosa	Amphipoea fucosa	1	oligo	Su
Amphipoea oculea	Amphipoea oculea	1	oligo	Su
Amphipyra berbera	Amphipyra berbera	0	poly	Su
Amphipyra pyramidea	Amphipyra pyramidea	0	poly	Au
Amphipyra tragopoginis	Amphipyra tragopoginis	0	poly	Au
Anaplectoides prasina	Anaplectoides prasina	0	poly	Su
Antitype chi	Antitype chi	0	poly	Au
Apamea anceps	Apamea anceps	0	oligo	Su
Apamea furva	Apamea furva	0	oligo	Su
Apamea illyria	Apamea illyria	0	oligo	Su
Apamea lithoxylaea	Apamea lithoxylaea	0	oligo	Su
Apamea sordens	Apamea sordens	0	oligo	Su
Apamea sublustris	Apamea sublustris	0	oligo	Su
Apamea unanimis	Apamea unanimis	0	oligo	Su
Apamea monoglypha	Apamea monoglypha	1	poly	Su
Apamea oblonga	Apamea oblonga	1	oligo	Su
Apamea scolopacina	Apamea scolopacina	1	poly	Su
Apamea crenata	Apamea crenata	2	oligo	Su
Apamea ophiogramma	Apamea ophiogramma	2	oligo	Su
Apamea remissa	Apamea remissa	2	oligo	Su
Aporophyla lutulenta	Aporophyla lutulenta	2	poly	Au
Archanara algae	Archanara algae	0	oligo	Au
Archanara sparganii	Archanara sparganii	0	mono	Su
Archanara dissoluta	Archanara dissoluta	1	mono	Au
Archanara geminipuncta	Archanara geminipuncta	1	oligo	Au
Arenostola phragmitidis	Arenostola phragmitidis	0	mono	Su
Autographa buraetica	Autographa buraetica	0	poly	Su
Autographa gamma	Autographa gamma	0	poly	Su
Autographa jota	Autographa jota	0	poly	Su
Autographa mandarina	Autographa mandarina	0	poly	Su
Autographa pulchrina	Autographa pulchrina	0	poly	Su
Axylia putris	Axylia putris	0	poly	Su
Blepharita satura	Mniotype satura	0	poly	Au
Caradrina morpheus	Caradrina morpheus	1	poly	Su
Catocala nupta	*Catocala nupta	0	poly	Au
Catocala promissa	*Catocala promissa	0	poly	Au
Catocala fraxini	*Catocala fraxini	1	mono	Su
Celaena leucostigma	Celaena leucostigma	2	poly	Su
Cerapteryx graminis	Cerapteryx graminis	0	oligo	Su
Cerastis leucographa	Cerastis leucographa	0	poly	Sp
Cerastis rubricosa	Cerastis rubricosa	0	poly	Sp
Charanyca trigrammica	Charanyca trigrammica	0	poly	Su
Chersotis cuprea	Chersotis cuprea	0	poly	Au

Chilodes maritima	Chilodes maritima	2	oligo	Su
Chortodes brevilinea	Protarchanara brevilinea	0	mono	Su
Chortodes elymi	Chortodes elymi	0	mono	Su
Chortodes extrema	Chortodes extrema	0	mono	Su
Chortodes fluxa	Chortodes fluxa	1	mono	Su
Chortodes pygmina	Sedina pygmina	1	poly	Au
Colocasia coryli	Colocasia coryli	1	poly	Su
Conistra rubiginosa	Conistra rubiginosa	0	oligo	SpAu
Conistra erythrocephala	Conistra erythrocephala	2	poly	SpAu
Conistra vaccinii	Conistra vaccinii	2	poly	SpAu
Cosmia pyralina	Cosmia pyralina	0	poly	Su
Cosmia trapezina	Cosmia trapezina	2	poly	Su
Craniophora ligustri	Craniophora ligustri	1	oligo	Su
Cryphia raptricula	Cryphia raptricula	1	oligo	Su
Cucullia absinthii	Cucullia absinthii	0	oligo	Su
Cucullia chamomillae	Cucullia chamomillae	0	oligo	Sp
Cucullia fraudatrix	Cucullia fraudatrix	0	mono	Su
Cucullia praecana	Cucullia praecana	0	oligo	Su
Cucullia umbratica	Cucullia umbratica	0	oligo	Su
Dasypolia templi	Dasypolia templi	0	poly	SpAu
Deltote bankiana	Deltote bankiana	0	oligo	Su
Deltote uncula	Deltote uncula	0	oligo	Su
Diachrysia chrysitis	Diachrysia chrysitis	0	poly	Su
Diachrysia tutti	Diachrysia tutti	0	poly	Su
Diarsia brunnea	Diarsia brunnea	0	poly	Su
Diarsia florida	Diarsia florida	0	poly	Au
Diarsia dahlii	Diarsia dahlii	1	poly	Su
Diarsia rubi	Diarsia rubi	1	poly	Sp
Diarsia mendica	Diarsia mendica	2	poly	Su
Dichonia aprilina	Dichonia aprilina	0	oligo	Au
Discestra trifolii	Discestra trifolii	1	poly	Su
Dypterygia scabriuscula	Dypterygia scabriuscula	0	oligo	Su
Emmelia trabealis	Emmelia trabealis	0	mono	Su
Enargia paleacea	Enargia paleacea	0	poly	Su
Eremobia ochroleuca	Eremobia ochroleuca	0	oligo	Su
Eucarta virgo	Eucarta virgo	0	poly	Su
Eugnorisma depuncta	Eugnorisma depunctum	0	poly	Su
Euplexia lucipara	Euplexia lucipara	0	poly	Su
Eupsilia transversa	Eupsilia transversa	1	poly	SpAu
Eurois occulta	Eurois occulta	1	poly	Su
Euxoa nigricans	Euxoa nigricans	0	poly	Su
Euxoa nigrofusca	Euxoa nigriofusca	0	poly	Su
Euxoa obelisca	Euxoa obelisca	0	poly	Su
Euxoa recussa	Euxoa recussa	1	poly	Su
Euxoa tritici	Euxoa tritici	1	poly	Su
Euxoa cursoria	Euxoa cursoria	2	poly	Su
Gortyna flavago	Gortyna flavago	0	poly	Au
Graphiphora augur	Graphiphora augur	0	poly	Su

Hada plebeja	Hada plebeja	1	poly	Su
Hadena bicruris	Hadena bicruris	0	oligo	Su
Hadena compta	Hadena compta	0	oligo	Su
Hadena confusa	Hadena confusa	0	oligo	Su
Hadena perplexa	Hadena perplexa	0	oligo	Su
Hadena rivularis	Sideridis rivularis	0	oligo	Su
Helicoverpa armigera	Helicoverpa armigera	0	poly	Su
Heliophobus reticulata	Heliophobus reticulata	0	poly	Su
Heliothis maritima	Heliothis maritima	0	oligo	Su
Heliothis viriplaca	Heliothis viriplaca	0	poly	Su
Herminia grisealis	*Herminia grisealis	0	poly	Su
Hoplodrina ambigua	Hoplodrina ambigua	0	oligo	Au
Hoplodrina blanda	Hoplodrina blanda	0	poly	Su
Hoplodrina octogenaria	Hoplodrina octogenaria	0	poly	Su
Hydraecia petasitis	Hydraecia petasitis	0	poly	Su
Hydraecia ultima	Hydraecia ultima	0	mono	Au
Hydraecia micacea	Hydraecia micacea	1	mono	Su
Hypena proboscidalis	*Hypena proboscidalis	0	mono	Su
Hypena rostralis	*Hypena rostralis	2	mono	SpAu
Ipimorpha subtusa	Ipimorpha subtusa	0	mono	Su
Lacanobia contigua	Lacanobia contigua	0	poly	Su
Lacanobia oleracea	Lacanobia oleracea	0	poly	Su
Lacanobia thalassina	Lacanobia thalassina	0	poly	Su
Lacanobia w-latinum	Lacanobia W-latinum	0	poly	Su
Lacanobia suasa	Lacanobia suasa	2	poly	Su
Lasionycta proxima	Lasionycta proxima	0	poly	Su
Laspeyria flexula	Laspeyria flexula	0	oligo	Su
Lithophane furcifera	Lithomoia furcifera	0	poly	Au
Lithomoia solidaginis	Lithophane solidaginis	1	poly	Au
Luperina testacea	Luperina testacea	1	oligo	Au
Lycophotia porphyrea	Lycophotia porphyrea	0	mono	Su
Lygephila pastinum	*Lygephila pastinum	0	oligo	Su
Macdunnoughia confusa	Macdunnoughia confusa	0	poly	Su
Macrochilo cribrumalis	*Macrochilo cribrumalis	0	poly	Su
Mamestra brassicae	Mamestra brassicae	1	poly	Su
Melanchra persicariae	Melanchra persicariae	1	poly	Su
Melanchra pisi	Melanchra pisi	1	poly	Su
Mesapamea didyma	Mesapamea secalella	2	oligo	Su
Mesapamea secalis	Mesapamea secalis	2	oligo	Su
Mesoligia furuncula	Mesoligia furuncula	1	oligo	Su
Mesoligia literosa	Mesoligia literosa	1	poly	Su
Mniotype adusta	Mniotype adusta	0	poly	Su
Moma alpium	Moma alpium	0	poly	Su
Mythimna albipuncta	Mythimna albipuncta	0	oligo	Su
Mythimna comma	Mythimna comma	0	oligo	Su
Mythimna conigera	Mythimna conigera	0	oligo	Su
Mythimna ferrago	Mythimna ferrago	0	oligo	Su
Mythimna impura	Mythimna impura	0	mono	Sp

Mythimna l-album	Mythimna l-album	0	oligo	Su
Mythimna obsoleta	Mythimna obsoleta	0	oligo	Su
Mythimna pudorina	Mythimna pudorina	0	mono	Su
Mythimna straminea	Mythimna straminea	0	oligo	Su
Mythimna flammea	Mythimna flammea	1	oligo	Su
Mythimna pallens	Mythimna pallens	1	oligo	Su
Naenia typica	Naenia typica	0	poly	Su
Noctua janthina	Noctua janthina	0	poly	Su
Noctua comes	Noctua comes	1	poly	Su
Noctua interjecta	Noctua interjecta	1	poly	Su
Noctua interposita	Noctua interposita	1	poly	Su
Noctua janthe	Noctua janthe	1	poly	Su
Noctua orbona	Noctua orbona	1	poly	Su
Noctua fimbriata	Noctua fimbriata	2	poly	Su
Noctua pronuba	Noctua pronuba	2	poly	Su
Nonagria typhae	Nonagria typhae	2	mono	Au
Ochropleura plecta	Ochropleura plecta	0	poly	Su
Oligia fasciuncula	Oligia fasciuncula	0	oligo	Su
Oligia versicolor	Oligia versicolor	0	oligo	Su
Oligia latruncula	Oligia latruncula	1	oligo	Su
Oligia strigilis	Oligia strigilis	1	oligo	Su
Orthosia cerasi	Orthosia cerasi	0	poly	Sp
Orthosia gracilis	Orthosia gracilis	0	poly	Sp
Orthosia opima	Orthosia optima	0	poly	Sp
Orthosia cruda	Orthosia cruda	1	poly	Sp
Orthosia populeti	Orthosia populeti	1	poly	Sp
Orthosia gothica	Orthosia gothica	2	poly	Sp
Orthosia incerta	Orthosia incerta	2	mono	Sp
Panolis flammea	Panolis flammea	0	mono	Su
Panthea coenobita	Panthea coenobita	0	oligo	Su
Papestra biren	Papestra biren	0	poly	Su
Paradrina clavipalpis	Paradrina clavipalpis	1	poly	Su
Parascotia fuliginaria	*Parascotia fuliginaria	0	oligo	Su
Parastichtis suspecta	Parastichtis suspecta	1	poly	Su
Parastichtis ypsillon	Parastichtis ypsillon	1	poly	Su
Pechipogo strigilata	*Polypogon strigilata	0	poly	Su
Peridroma saucia	Peridroma saucia	2	poly	Au
Phlogophora meticulosa	Phlogophora meticulosa	1	poly	SpAu
Photedes minima	Photedes minima	0	oligo	Su
Plusia festucae	Plusia festucae	0	oligo	Au
Plusia putnami	Plusia putnami	0	oligo	Su
Polia bombycina	Polia bombycina	0	poly	Su
Polia nebulosa	Polia nebulosa	0	poly	Su
Polymixis polymita	Polymixis polymita	0	poly	Au
Protodeltote pygarga	Protodeltote pygarga	0	oligo	Su
Pseudeustrotia candidula	Pseudeustrotia candidula	0	poly	Su
Pyrrhia umbra	Pyrrhia umbra	0	poly	Su
Rhizedra lutosa	Rhizedra lutosa	1	mono	Au

Rhyacia simulans	Rhyacia simulans	0	poly	Su
Rivula sericealis	*Rivula sericealis	1	oligo	Su
Rusina ferruginea	Rusina ferruginea	0	poly	Su
Schrankia costaestrigalis	*Schrankia costaestrigalis	0	poly	Su
Scoliopteryx libatrix	*Scoliopteryx libatrix	0	oligo	SpAu
Sedina buettneri	Sedina buettneri	0	mono	Au
Shargacucullia lychnitis	Shargacucullia lychnitis	0	mono	Su
Sideridis albicolon	Sideridis reticulata	0	poly	Su
Simyra albovenosa	Simyra albovenosa	1	oligo	Su
Spaelotis ravida	Spaelotis ravida	0	poly	Su
Spodoptera exigua	Spodoptera exigua	0	poly	Su
Staurophora celsia	Staurophora celsia	0	oligo	Au
Syngrapha interrogationis	Syngrapha interrogationis	0	oligo	Su
Thalpophila matura	Thalpophila matura	1	oligo	Su
Tholera cespitis	Tholera cespitis	0	oligo	Au
Tholera decimalis	Tholera decimalis	0	oligo	Au
Trachea atriplicis	Trachea atriplicis	0	oligo	Su
Trisateles emortualis	*Trisateles emortualis	0	poly	Su
Tyta luctuosa	Tyta luctuosa	0	mono	Su
Xanthia togata	Xanthia togata	0	poly	Au
Xanthia gilvago	Xanthia gilvago	1	mono	Au
Xanthia icteritia	Xanthia icteritia	1	poly	Au
Xanthia ocellaris	Xanthia ocellaris	1	mono	Au
Xanthia aurago	Xanthia aurago	2	poly	Au
Xestia baja	Xestia baja	0	poly	Su
Xestia rhomboidea	Xestia rhomboidea	0	poly	Su
Xestia sexstrigata	Xestia sexstrigata	0	poly	Su
Xestia triangulum	Xestia triangulum	0	poly	Su
Xestia c-nigrum	Xestia c-nigrum	1	poly	Su
Xestia ashworthii	Xestia ashworthii	2	poly	Su
Xestia xanthographa	Xestia xanthographa	2	poly	Au
Xylena exsoleta	Xylena exsoleta	0	poly	SpAu
Xylena vetusta	Xylena vetusta	0	poly	SpAu
Zanclognatha tarsipennalis	*Herminia tarsipennalis	0	poly	Su

SUPPORTING REFERENCES

1. Karsholt O., Razawski J. 1996 *The Lepidoptera of Europe - a distributional checklist*. Stenstrup, Denmark, Apollo Books.
2. Karsholt O., Stadel Nielsen P. 2013 *Revideret fortegnelse over Danmarks Sommerfugle: Revised Checklist of the Lepidoptera of Denmark*. København, Lepidopterologisk Forening, København.
3. Betzholtz P.-E., Pettersson L.B., Ryrholm N., Franzén M. 2013 With that diet, you will go far: trait-based analysis reveals a link between rapid range expansion and a nitrogen-favoured diet. *Proc R Soc B* **280**. (doi:10.1098/rspb.2012.2305).
4. Svensson I. 1993 *Lepidoptera Calender*. Stockholm, Sweden, Hans Hellberg.
