# Supplementary material for: Variable coloration is associated with dampened population fluctuations in noctuid moths
Source: Proc Biol Sci. 2015 Jun 7;282(1808):20142922. doi: 10.1098/rspb.2014.2922 (PMC4455791; doi:10.1098/rspb.2014.2922)
Supplement: ESM Supporting Results 2015 04 14.pdf [file rspb20142922supp2.pdf]

Electronic Supplementary Material ESM (for publication as supporting information on the Proc R Soc London B website)

Variable coloration is associated with dampened population fluctuations in noctuid moths

Anders Forsman, Per-Eric Betzholtz, Markus Franzén

[1] Author for correspondence E-mail: anders.forsman@lnu.se

SUPPORTING RESULTS

The systematics and phylogenetic relationships of noctuid moths is subject to debate and under revision. For the results reported in the main text we used the systematics of Karlsholt and Razowski [1]. According to a more recent systematic treatment [2] some of the species in our data set have been renamed and assigned to a different genera, and 15 species in our data set have been moved from family Noctuidae to family Erebidae (**Table S1**). To evaluate whether our results and conclusions are influenced to any important degree by the systematic arrangements of species, we analysed our data also using the more recent systematics of Karsholt and Stadel Nielsen [2] and we did this in two ways, both including and omitting the 15 Erebidae species. The rearrangement of some species and the reduction in sample size when omitting Erebidae species, sensu Karsholt & Stadel Nielsen [2], resulted in that the exact parameter values of the statistical tests changed somewhat but these minor changes did not in any way influence the results or conclusions regarding abundance and stability in relation to colour pattern variability (cf. Results in main text with Results presented below).

Results based on systematics according to Karsholt and Stadel Nielsen 2013 [2] including the Erebidae species. Mean abundance varied significantly among noctuid genera and among the three levels of colour pattern diversity (general linear mixed model, GLMM, analysis, implemented using the procedure MIXED in SAS, applied to log transformed species means computed over the 11-year sampling period, random effect of genus: estimate = 0.0633 ± 0.0275SE, χ^2 = 6.2, df = 1, $0.01 < p < 0.025$, Z = 2.30, P = 0.0107; fixed effect of colour pattern: $F_{2,240}$ = 8.92, P = 0.0002).

Comparisons of among-year variability in abundance (estimated as within-species coefficient of variation CV) uncovered that species with non-variable colour patterns had greater fluctuations in abundance than did species with variable and highly variable colour patterns (GLMM, fixed effect of colour: $F_{2,243}$ = 5.23, P = 0.006)

There was no indication that species having more variable colour patterns as adults utilized a greater (or lower) diversity of host plant species (Mantel-Haenszel Chi-Square = 0.58, df = 1, P = 0.45, N = 246 species). The pattern of higher average abundance and reduced between year fluctuations in species with more variable colour patterns remained statistically significant when the potentially confounding effects of degree of larval host-plant specificity were adjusted for (mean abundance: fixed effect of colour pattern: $F_{2,239}$ = 8.88, P = 0.0002; fixed effect of larval niche breadth: $F_{2,197}$ = 0.66, P = 0.52; CV of abundance: fixed effect of colour pattern: $F_{2,241}$ = 5.16, P = 0.0064; fixed effect of larval niche breadth: $F_{2,141}$ = 2.90, P = 0.057).

Results based on systematics according to Karsholt and Stadel Nielsen 2013 [2] after omitting those 15 species now assigned to family Erebidae. Mean abundance varied significantly among noctuid genera and among the three levels of colour pattern diversity (general linear mixed model, GLMM, analysis, implemented using the procedure MIXED in SAS, applied to log transformed species means computed over the 11-year sampling period, random effect of genus: estimate = 0.0668 ± 0.0305SE, $\chi^2 = 6.2$, $df = 1$, $0.01 < p < 0.025$, $Z = 2.20$, $P = 0.0141$; fixed effect of colour pattern: $F_{2,225} = 8.19$, $P = 0.0004$).

Comparisons of among-year variability in abundance (estimated as within-species coefficient of variation CV) uncovered that species with non-variable colour patterns had greater fluctuations in abundance than did species with variable and highly variable colour patterns (GLMM, fixed effect of colour: $F_{2,228} = 5.75$, $P = 0.0037$)

There was no indication that species having more variable colour patterns as adults utilized a greater (or lower) diversity of host plant species (Mantel-Haenszel Chi-Square = 1.77, $df = 1$, $P = 0.18$, $N = 231$ species). The pattern of higher average abundance and reduced between year fluctuations in species with more variable colour patterns remained statistically significant when the potentially confounding effects of degree of larval host-plant specificity were adjusted for (mean abundance: fixed effect of colour pattern: $F_{2,224} = 7.91$, $P = 0.0005$; fixed effect of larval niche breadth: $F_{2,180} = 0.52$, $P = 0.60$; CV of abundance: fixed effect of colour pattern: $F_{2,226} = 5.34$, $P = 0.0054$; fixed effect of larval niche breadth: $F_{2,226} = 2.17$, $P = 0.12$).

SUPPORTING REFERENCES

1. Karsholt O., Razawski J. 1996 *The Lepidoptera of Europe - a distributional checklist*. Stenstrup, Denmark, Apollo Books.
2. Karsholt O., Stadel Nielsen P. 2013 *Revideret fortegnelse over Danmarks Sommerfugle: Revised Checklist of the Lepidoptera of Denmark*. København, Lepidopterologisk Forening, København.
